# Plastid Transformation: How Does it Work? Can it Be Applied to Crops? What Can it Offer?

**DOI:** 10.3390/ijms21144854

**Published:** 2020-07-09

**Authors:** Yihe Yu, Po-Cheng Yu, Wan-Jung Chang, Keke Yu, Choun-Sea Lin

**Affiliations:** 1 College of Forestry, Henan University of Science and Technology, Luoyang 471023, China; yuyihe@haust.edu.cn (Y.Y.); yukeke918@163.com (K.Y.); 2 Department of Chemistry, Temple University, Philadelphia, PA 19122, USA; tug55568@temple.edu; 3 Perelman School of Medicine, University of Pennsylvania, Philadelphia, PA 19104, USA; wanjungc@upenn.edu; 4 Agricultural Biotechnology Research Center, Academia Sinica, Taipei 115, Taiwan

**Keywords:** chloroplast, SWNTs, morphogenic regulators, CRISPR, protoplast regeneration

## Abstract

In recent years, plant genetic engineering has advanced agriculture in terms of crop improvement, stress and disease resistance, and pharmaceutical biosynthesis. Cells from land plants and algae contain three organelles that harbor DNA: the nucleus, plastid, and mitochondria. Although the most common approach for many plant species is the introduction of foreign DNA into the nucleus (nuclear transformation) via Agrobacterium- or biolistics-mediated delivery of transgenes, plastid transformation offers an alternative means for plant transformation. Since there are many copies of the chloroplast genome in each cell, higher levels of protein accumulation can often be achieved from transgenes inserted in the chloroplast genome compared to the nuclear genome. Chloroplasts are therefore becoming attractive hosts for the introduction of new agronomic traits, as well as for the biosynthesis of high-value pharmaceuticals, biomaterials and industrial enzymes. This review provides a comprehensive historical and biological perspective on plastid transformation, with a focus on current and emerging approaches such as the use of single-walled carbon nanotubes (SWNTs) as DNA delivery vehicles, overexpressing morphogenic regulators to enhance regeneration ability, applying genome editing techniques to accelerate double-stranded break formation, and reconsidering protoplasts as a viable material for plastid genome engineering, even in transformation-recalcitrant species.

## 1. Introduction

The plastids of algae and land plants are semi-autonomous organelles with their own genomes, complete with transcription and translation machinery [1] that arose from endosymbiosis between a eukaryotic ancestor and a photosynthetic cyanobacterium [2]. The plastid genome is a circular double-stranded DNA molecule that encodes 100–250 genes and is maternally inherited in angiosperms [3]. The size of chloroplast genomes varies between species, ranging from 107 kbp (Cathay silver fir, *Cathaya argyrophylla*) to 218 kbp (Geranium, *Pelargonium* spp.). The plastid genome is present in many copies inside the organelle and is protected from gene silencing pathways that typically lower transgene expression, thereby allowing high levels of foreign protein to accumulate, achieving 5–40% total soluble protein (TSP) [4] and up to 70% of total soluble protein in tobacco (*Nicotiana tabacum*) [5,6,7]. An even higher protein yield (>75% TSP) was recently achieved in tobacco transplastomic plants expressing a hyper-thermostable form of β-glucosidase cloned from the hyperthermophilic bacterium *Pyrococcus furiosus* [8]. Compared to nuclear transformation, plastid transformation has many advantages. First, the maternal inheritance of the chloroplast genome prevents genes from escaping through pollen grains in most plants and thus reduces the spread of transgenes in the environment and avoids issues with Genetically Modified Organisms (GMOs) [9]. Second, the multiple copies of the plastid genome contained within the organelle results in the high expression of foreign genes and the accumulation of the encoded proteins [10]. Third, since plastid transformation involves homologous recombination (HR) of the transgene into a so-called neutral site, the method largely avoids gene silencing caused by position effects [11]. Fourth, the chloroplast employs a prokaryotic gene expression system and allows the easy and simultaneous expression of polycistronic genes [12]. Last, the stacking of multiple transgenes can be performed in a single and highly efficient transformation event [13]. Plastid transformation is, therefore, gaining traction [14].

The main crop species used for plastid transformation are in the Solanaceae, including tobacco, potato (*Solanum tuberosum*), tomato (*S. lycopersicum*), eggplant (*S. melongena*), and pepper (*Capsicum annuum*). Plastid transformation has also been reported in other important crop species, including soybean (*Glycine max*, Fabaceae), lettuce (*Lactuca sativa*, Asteraceae), cauliflower (*Brassica oleracea* var. *botrytis*, Brassicaceae), cotton (*Gossypium* spp., Malvaceae), carrot (*Daucus carota*, Umbelliferae), bitter squash (*Momordica charantia*, Cucurbitaceae) and rice (*Oryza sativa*, Poaceae) (Table 1). To date, the plastids of over 20 flowering plants have been transformed [15] (Table 1). In addition to the crops mentioned above, recent successes in plastid transformation have been reported in the plant species bitter melon [16], and the medicinal plant sweet wormwood (*Artemisia annua*) [17] and licorice weed (*Scoparia dulcis*) [18,19] (Table 1).

Based on these successful cases, plastid transformation should be applicable to many plant families, whether they are monocots or dicots. However, plastid transformation remains much more challenging than nuclear transformation and is not as widespread in plant research. Furthermore, Bock [7] raised the issue of reproducibility of plastid transformation, as the plastids of fewer than ten species have been demonstrably transformed in at least two independent reports since 1988, the year the first example of plastid transformation was published in the unicellular green alga Chlamydomonas (*Chlamydomonas reinhardtii*) [7,40]. Major critical points limiting current plastid transformation attempts are 1) the method of DNA delivery, 2) homologous recombination efficiency and 3) methods for efficient selection and robust regeneration of transformants. In this review, we will address each of these issues and will highlight recent innovative technologies and strategies in plastid transformation. We hope to give readers a new perspective on the potential of plastid transformation with concrete examples, setting the stage for plastid transformation in more plant species in the future. 

## 2. How to Transform a Chloroplast in Three Steps

Plastid transformation can be divided into three steps: first, foreign DNA is delivered to cells of an explant [41]. Second, the foreign DNA is inserted into the chloroplast genome through homologous recombination at a predetermined and precise location. Third, candidate transformants are repeatedly screened on selection medium until the wild-type genome is eliminated (a state known as homoplasmy) [9]. Positive explants are then regenerated into stable transgenic plants.

### 2.1. Robust Methods for DNA Delivery into the Chloroplast

The two most common methods for introducing foreign DNA into chloroplasts are biolistic transformation [9] and polyethylene glycol (PEG)-mediated transfection [42]. Biolistics delivers particles coated with DNA into plant cells by high-speed bombardment through a gene gun or a particle delivery system. This method can be applied to various plants by adjusting bombardment parameters such as distance to the target tissue, chamber vacuum pressure, particle size and DNA: particle ratios, to accommodate variations in leaf texture. The PEG-mediated plastid transformation method works on plant cells from which the cell wall has been removed (protoplasts). The co-culture of protoplasts in the presence of PEG vesicles loaded with plasmid DNA allows DNA uptake by protoplasts, leading to the integration of foreign DNA into the plastid genome [42]. Although the PEG method requires the enzymatic digestion of tissues to release protoplasts, it is a more economical procedure because it does not rely on a specialized and expensive delivery system.

A new strategy for plastid transformation via nanoparticles was recently introduced [43]. This method allows DNA to be delivered to chloroplasts simply through single-walled carbon nanotubes (SWNTs) without the need for additional instruments or protoplast isolation, or the aid of chemical reagents. Nanocarriers for transformation consisted of chitosan-complexed single-walled carbon nanotubes (CS–SWNTs). These nanotubes are positively charged and can, therefore, carry negatively charged plasmid DNA via electrostatic interactions; the resulting DNA-SWNT conjugate may easily enter leaf mesophyll cells by infiltration using a syringe from stomatal pores. Once the DNA-SWNT conjugate passes through the leaf surface and enters the mesophyll, it will eventually be trafficked to the chloroplast bilayers by way of lipid exchange envelope penetration [43]. 

The power of this delivery strategy lies in the fact that the DNA can be selectively released in the chloroplast due to differences in pH within the cell. The acidic cytosol (around pH 5.5) leaves the DNA tightly bound to chitosan; by contrast, the carriers tend to unload the DNA inside the chloroplast due to its weakly alkaline environment (~pH 8.0). This preferential release accomplishes the selective release of DNA at its intended target site. A similar strategy may also be applied to protoplasts by effectively replacing PEG with nanotubes [43]. Indeed, DNA alone does not have the ability to penetrate plant cells in the absence of the SWNT carrier, as demonstrated with protoplasts co-cultured with DNA only. Notably, the efficiency of DNA entrance depends on the zeta potential and hydrodynamic radius of SWNTs rather than the concentration of plasmid DNA. Using a reporter construct carrying the *Yellow Fluorescent Protein* gene (*YFP*), YFP fluorescence was detected after 24 hr incubation of protoplasts with a DNA:SWNT mixture in a 1:6 ratio, and the YFP signal largely coincided with chloroplasts. However, no fluorescence was detected when the DNA:SWNT ratio was increased to 1:1. Excess DNA, bound to SWNTs, will neutralize their surface charge, which will not only reduce the entry of DNA-SWNT conjugates but also increase their instability, resulting in a partial or complete loss of membrane crossing [43].

Using the same YFP reporter, the method was further tested on living plants, in the form here of four-week old arugula (*Eruca sativa*) plants. The authors tested different DNA:SWNT ratios (1:1, 1:3 and 1:6); not surprisingly, based on the reasoning provided above, only ratios of 1:3 and 1:6 expressed YFP. Transient YFP expression reached its peak 48 h after the initial infiltration. An impressive aspect of the study was the low amounts of DNA (~20 ng) required for successful delivery and expression of plasmid DNA to chloroplasts, far less than for PEG-mediated transformation (20–50 μg) or biolistics (5 μg) [44,45,46].

This article demonstrated the delivery and expression of a transgene to chloroplasts in five different plant species, including four mature living plants and isolated protoplasts, which indicates the potential of this nanoparticle delivery method for widespread application [43]. Recently, another method by Santana et al. used a chloroplast signal peptide as a guide to delivering nanomaterials loaded with chemicals into Arabidopsis chloroplasts [47]. Tagged with a 14-amino acid guide peptide, the cargo was sent to the translocons at the outer and inner membrane of chloroplasts (TOC/TIC) and was transported into the chloroplast stroma. This, therefore, provides a targeted way to deliver materials into plastids via a biorecognition motif [47]. In addition to biolistics and PEG-mediated delivery methods, carbon nanotubes and biorecognition peptides now join the team of plastid transformation tools and combine the advantages of low DNA amounts and high delivery accuracy.

However, all SWNT tests were based on transient expression using a single plasmid carrying flanking regions from switchgrass (*Panicum virgatum*) [43]. Although the YFP signal indeed originated from the chloroplast and thus demonstrated the trafficking function of SWNTs to the organelle, homologous recombination within the chloroplast genome remains to be confirmed. In the case of the signal peptide delivery technique [47], the chemical cargo was the subject of the test and not DNA. Therefore, the ability and the efficiency of stable transformation through these two methods remain to be determined. 

### 2.2. Harnessing Homologous Recombination in Chloroplasts

Homologous recombination is a crucial step after DNA delivery into the chloroplast that determines the subsequent success of the transformation. The frequency of HR events is highest when the foreign DNA carries a sequence of at least 121 bp that is identical to the target integration site [48]. Careful consideration should be given to the choice of promoter and regulatory elements (5′ and 3′ untranslated regions), as well as the insertion site in the plastid genome, to maximize transformation efficiency, as shown in Table 2. The *psbA* promoter, from the plastid-encoded photosystem II protein D1 precursor, was first used over 30 years ago in Chlamydomonas and still appears to be the best position in which to insert a target gene, as the *psbA* gene product is the most highly translated plastid protein [49]. Boynton, Gillham, and colleagues first achieved plastid transformation of Chlamydomonas with this plastid promoter in 1988 [40]; Svab, Hajukiewicz and Maliga followed, in 1990, with the first report of plastid transformation in tobacco, although they used a different fragment of chloroplast DNA in their target plasmid [50]. Plastid transformation has since been reported in many flowering plants, such as tobacco [50,51,52], Arabidopsis [53,54], potato [55], rice [56], rapeseed [57], and tomato [58]. A protocol for plastid transformation of an elite rapeseed cultivar (*B. napus* L.) has been developed [27]. The highest protein yields were accomplished by using the *rrn* promoter from the plastid rRNA operon, as reported in several reviews [7,8,59,60]. 

### 2.3. Selection Methods and Regeneration Protocols for Transplastomic Cells and Plants

Ideally, plant species commonly used for plastid transformation, such as tobacco, should have a well-developed tissue culture system and high regeneration potential. It is therefore generally considered that plastid transformation is confined to crops that fulfill these two criteria. Different plant species, or even different cultivars from the same species, require specific tissue culture conditions. Protocols for in vitro culture often take time to set up, including the optimization of the growth temperature, humidity, the composition of the culture medium, to name a few variables. Plastid transformation studies and their applications are thus still largely restricted to certain plant species like rice, tobacco, and lettuce.

Although plant tissue culture conditions vary extensively, the selection procedure for screening homoplasmic plants are quite similar and have not changed much over the past decades [60]. Several antibiotics have been used in plastid transformation [15,61]. For example, the expression of *Neomycin Phosphotransferase* (*nptII*) or *Aminoglycoside 3′-Phosphotransferase* (*aphA6*) confers resistance to kanamycin [31,62], while the expression of *Bialaphos Resistance* (*bar*) provides resistance to the herbicide glyphosate in transgenic plants. However, the gene *Streptomycin 3′-Adenylyltransferase* (*aadA*), a spectinomycin and streptomycin resistance gene, remains the most commonly used marker for plastid transformation [15,60]. Spectinomycin inhibits plastid protein translation by binding to chloroplast ribosomes [63,64]. At least in Chlamydomonas and tobacco, mutations in the plastid ribosomal subunit targeted by spectinomycin confer resistance to the antibiotic. In fact, the first report of plastid transformation in tobacco used a plastid DNA fragment carrying such a mutation to demonstrate transformation potential and act as a selectable marker at the same time [50].

Parker et al. reported that one spectinomycin tolerance strategy in Arabidopsis involves Acetyl-Coa Carboxylase 2 (ACC2) [65,66]. Indeed, seedlings cannot develop beyond the cotyledon stage under spectinomycin selection when they have a functional copy of the nuclear *ACC2* gene, thereby limiting selection efficiency in Arabidopsis plastid transformation. Based on these results, Yu et al. went on to use an *acc2* loss of function Arabidopsis mutant (SALK_148966C) to test the effectiveness of particle bombardment with a plasmid encoding an aadA-GFP fusion [25]. Transformation efficiency increased around 100-fold in the *acc2* mutant background. Ruf et al. later used clustered regularly interspaced short palindromic repeats (CRISPR)/Cas9-mediated genome editing to inactivate the *ACC2* locus and create a recipient line for plastid transformation. Transformants in this *acc2* mutant background grew to maturity and produced seeds, as the loss of ACC2 function is not accompanied by visible growth phenotypes [67]. These reports may, therefore, open an avenue for routine engineering of the plastid genome in Arabidopsis, and provide valuable information for plastid transformation in other, more recalcitrant species.

## 3. Can Plastid Transformation Work in Crops? CRISPR-Cas, Morphogenic Regulators, and Protoplast Regeneration Can Help

Although tobacco is an excellent species for plastid transformation, its leaves are not edible, so any recombinant protein produced in the chloroplast needs to be purified before use. By contrast, plastid transformation of vegetable and fruit plants would offer significant advantages for the production of edible vaccines. Fruits are easier to store and transport relative to leafy vegetables like lettuce. The establishment of plastid transformation in species that bear fruit suitable for human consumption is a hot research topic. 

For plastid transformation, the formation of double-strand DNA breaks (DSBs) in the chloroplast genome is a critical factor for HR [56], which might be stimulated at the chosen editing site by the use of the CRISPR nuclease Cas9 or transcription activator-like effector nucleases (TALENs). The CRISPR/Cas9 system allows for precise genome editing by guide RNAs (gRNAs) that direct the Cas9 nuclease to a target site. To increase plastid transformation efficiency, Yoo et al. [68] applied genome editing in Chlamydomonas chloroplasts by introducing two plasmids: one plasmid carried both a gRNA and a Cas9 expression cassette, while the other plasmid bore the donor DNA fragment for integration at the DSB site created by the action of Cas9 and the gRNA. Both plasmids were transformed into the alga by biolistics; cell lysates from individual transformants were screened by PCR 28 days after bombardment. When Cas9/gRNA was placed under the control of the strong chloroplast *psaA* promoter, two transformants out of 20 had the donor DNA at the intended integration site. By contrast, no HR events were observed when Cas9/gRNA were expressed from the weaker plastid *psbD* promoter, or when the first plasmid carried the gRNA but not Cas9. These results indicate that DNA breaks indeed promote donor DNA integration. Although the CRISPR-Cas approach has not yet been applied to plastid transformation in plants, a sequential transformation method was used to generate nuclear HR transgenic lines in Arabidopsis [69]. Two transgenic lines expressing Cas9 were used as parental lines for stable transformation with a second construct carrying the sgRNA and donor DNA. Using this sequential transformation approach, transgenic plants with HR in the target site of the nuclear genome were successfully identified [69]. Similarly, incorporating Cas9 and gRNAs into plastid transformation protocols should accelerate DSB formation and raise the frequency of HR, as high expression of Cas9 would enhance the likelihood of HR in transgenic plants. 

TALENs constitute another potential strategy for site-specific gene modification and have been widely used in algae and flowering plants to generate transformants with desired traits [70,71,72]. For example, Li et al. [73] co-bombarded a plasmid carrying a TALEN construct and a plasmid carrying an HR DNA fragment in rice using biolistics. The transformation efficiency of the TALEN-assisted group was twice as high as that of a group transformed only with the HR fragment-containing plasmid. Together, these recent results provide a convincing foundation for the application of genome editing by Cas9 or TALENs in plastid transformation.

Another limiting factor for any plastid transformation effort is the regeneration efficiency during tissue culture. Harnessing growth-related genes to promote plant growth is not a new concept: in fact, the idea of affecting plant morphogenesis to recover transformants can be traced back to the 1980s [74]. Previous studies have shown that overexpressing morphogenic genes can increase nuclear transformation rates and enhance regeneration ability [75,76]. To date, several morphogenic genes (*Baby boom* (*BBM*) and *Wuschel2* (*WUS2*)) have been successfully used in nuclear transformation [76,77,78]. This strategy has not yet been applied to plastid transformation and is worth exploring as an approach to support plastid transformation in more species, especially those plants that are recalcitrant to transformation or are only marginally transformable.

Thanks to the advances in transformation technology, research facilities and purity of chemicals, protoplasts might be an option worth (re)-considering for plants that are not amenable to transformation by biolistics or when morphogenic regulators are ineffective. Protoplast regeneration was reported as early as the 1970s [79]. Unfortunately, protoplast regeneration is perceived by many as being hard to establish and labor-intensive and is always considered as a last resort, when it is even considered. This impression might stem from the poor quality of chemicals and laboratory conditions in the early days. With the development of more effective tissue culture protocols and the evolution of laboratory facilities, protoplast regeneration may be established in any plant tissue culture laboratory with minimal effort [44,80]. This technique, in fact, offers many advantages: there is no need for expensive instruments and consumables cost is low. Protoplast transformation may even be considered as being more efficient than other transformation techniques for flowering plants. Multiple rounds of particle bombardment are typically required on hundreds of leaves to obtain transformants, while protoplasts derived from only two leaves and transformed with PEG vesicles loaded with target DNA can produce positive clones in a single experiment [42]. Numerous articles have reported success in PEG-mediated protoplast transformation and regeneration [42,80,81,82]; some studies also demonstrated that protoplast transformation supported the transformation of non-transformable plants and increased the expression of a foreign (target) gene. In cauliflower, no transformants were obtained by biolistics, but DNA uptake was achieved by protoplasts in the presence of PEG, although the transformation efficiency was not high [82]. In potato, high activity levels were detected for a reporter carrying the *beta-Glucuronidase* (*GUS*) gene transformed into protoplasts using the PEG method. By contrast, transgenic plants obtained by biolistics showed variable GUS activity levels, and truncated RNA species were detected in plants with low GUS activity [81]. This phenomenon of transgene silencing in lines generated by biolistics is frequently reported, in contrast to Agrobacterium- or PEG-mediated transformation in rice and barley [83,84]. 

To date, our laboratory has successfully transformed protoplasts with the PEG-mediated method and regenerated whole plants for tobacco and *N. benthamiana*, tomato, wild tomato (*S. peruvianum*), rapid-cycling Brassica and Arabidopsis. Taking tobacco as an example, it only took our laboratory about two years to establish the entire procedure, from protoplast isolation and CRISPR/Cas9 transformation by PEG-mediated transfection to protoplast regeneration [80]; by no means a short time frame, but definitely manageable and attainable. With the incorporation of the latest tools such as SWNTs delivery or morphogenic regulators, we believe that our platform can attain a wider application by reaching more crops or when applied to plastid transformation. 

## 4. What Needs Can Plastid Transformation Fill?

Plastid transformation was developed over three decades ago, and numerous transplastomic algae and flowering plants have been created successfully. What can these transplastomic plants do? Here, we review the plastid transformation literature to give our readers an understanding of the potential applications.

Engineering chloroplasts with desired agronomic traits has garnered interest in recent years. For instance, expressing a bacterial *4-Hydroxyphenylpyruvate Dioxygenase* (*HPPD*) gene in tobacco or soybean chloroplasts conferred enhanced herbicide resistance [30], while expression of *Betaine Aldehyde Dehydrogenase* (*BADH*) in carrot chloroplasts provided strong salt tolerance [39]. As mentioned earlier, tobacco remains by far the most suitable species for plastid transformation, although the technique has also been successfully applied to other species such as tomato [85,86], potato [37], maize [87], sugar beet [24], cotton [31] and wheat [88]. For example, the simultaneous expression of protease inhibitors and chitinase in transplastomic tobacco plants conferred resistance to multiple biotic and abiotic stresses [89]. Multiple economic and agronomic traits of interest have been engineered into chloroplasts, including resistance to cold, drought, insects or herbicides as well as salt tolerance [30,90,91,92]. Herbicide resistance is perhaps one of the most notable traits in plastid transformation. Plants resistant to the herbicide glyphosate (commercialized as Roundup) were generated by introducing the *5-Enolpyruvylshikimate-3-Phosphate Synthase* (*EPSPS*) gene into the tobacco plastid genome, which encodes an enzyme that detoxifies glyphosate [4]. Agronomic characters obtained by the engineering of the chloroplast genome are listed in Table 3; most transformations relied on biolistics, although these largely predate the publication of the SWNTs tool, which may provide a new opportunity for scientists thanks to its low cost and ease of use.

### 4.1. Antigen Vaccines and Protein-based Drugs

Globally, the number of individuals suffering from diabetes is expected to rise from 170 million in 2000 to a projected 366 million by 2030 [114]. More than 90% of the global population cannot afford the cost of insulin [3,115]. Protein-based drugs such as insulin are expensive because they are produced in yeast fermentation systems and later kept in cold storage, but the final pure product still has a short shelf-life [116]. However, protein-based drugs produced in transplastomic plants may solve many of the associated issues without raising costs or compromising drug efficacy [3]. Many vaccine antigens and biopharmaceuticals have been successfully produced from the chloroplasts of flowering plants. 

The induction of insulin production in human subjects is an attractive alternative to daily insulin injections. Exendin-4 (EX4), an analog of the peptide hormone Glucagon-like peptide, was expressed in tobacco chloroplasts, fused to the Cholera toxin B (CTB) subunit to facilitate delivery by crossing the intestinal epithelium. Lyophilized tobacco leaf extracts increased insulin production levels in mice without inducing hypoglycemia, even when a 5000-fold excess dose of CTB-EX4 was delivered orally [117]. In addition, the accumulation of human interferon-gamma in tobacco chloroplasts reached 0.42% of total soluble protein [118]. Unlike microorganisms, plant chloroplasts can perform post-translational modifications of protein-based drugs and promote their proper folding: phosphorylation, amidation, and disulfide bond formation [119]. 

Human papillomavirus (HPV) is a cause of cervical cancer, which kills over 250,000 women each year. Protein E7 from HPV type 16 (HPV-16 E7) is an attractive anti-cancer vaccine antigen that has been expressed in tobacco via plastid transformation or transient expression [120,121,122]. The plant-produced proteins successfully induced an immune response and mediated tumor regression in the murine model. Using the transient transfection system with Agrobacterium LBA4404 and the pBIN-NSs vector containing the TSWV NSs silencing suppressor gene, the E7 protein fused with Zera® was expressed only at levels ranging from 0.1–6 g/kg [121]. Via plastid transformation, E7 could reach 0.1% TSP in transplastomic plants [122]. Notably, E7-potato virus X coat protein fusion proteins accumulated to levels around five times higher than the unfused E7 [120,122]. 

Human coagulation factors made from plants have also been shown to improve immune tolerance in hemophilia murine and canine models [23,123]. In addition, high-level expression of vaccine antigens and therapeutic proteins has been achieved in plant chloroplasts (leaves and roots) or chromoplasts (fruits) for antigens associated with the plague, tetanus, human immunodeficiency virus (HIV), cholera, malaria, Alzheimer’s disease and hemophilia [123,124,125,126,127,128,129,130]. Table 4 provides a partial list of vaccine antigens and drug proteins expressed in the chloroplast. Although high levels of protein expression are desirable for chloroplast production of protein-based drugs, excessive expression of foreign proteins may poison host plants. However, the chloroplast of the unicellular green alga Chlamydomonas largely possesses the same machinery necessary for folding and assembling complex eukaryotic proteins, as that of flowering plants and tolerates the accumulation of eukaryotic toxins [20]. Protein-based drugs and vaccine antigens produced in Chlamydomonas chloroplasts are shown in Table 5 [20,131,132,133,134,135,136,137].

### 4.2. Industrial Enzymes and Biomaterials

The chloroplast genome has been repeatedly engineered to produce industrial enzymes and biomaterials. Polyhydroxyalkanoates (PHAs) are a large class of biodegradable polyesters biopolymers naturally synthesized by many microorganisms that can be used as an alternative to petroleum-based plastics [137]. The first described and most well-studied PHA is polyhydroxybutyrate (PHB). Various systems have been adapted for the production of PHB, including microbial cells and various plant tissues. To date, however, the highest level of PHB accumulation was achieved in tobacco plastids, with levels of 18.8% of dry weight (DW). The tobacco system was based on an operon extension strategy to synthesize high PHB levels by introducing a bacterial operon, consisting of three genes encoding enzymes necessary for PHB biosynthesis, into the tobacco chloroplast genome [145]. The high amounts of PHB produced in this system stems from the high flux of the PHB biosynthetic precursor acetyl-CoA released during fatty acid biosynthesis [146]. Typical examples of industrial enzymes and biomaterials obtained through plastid transformation are given in Table 6 [8,97,145,147,148,149,150,151].

### 4.3. Phytoremediation

Mercury (Hg), especially in its organic form, is a highly toxic pollutant that affects humans, animals, and plants alike. At present, phytoremediation is a cost-effective method to remove heavy metals from contaminated soils by using plants to clean up contaminated environments by taking up the desired pollutant [152]. In plants, Hg mainly targets chloroplasts, where it impairs electron transport and photosynthesis. Therefore, chloroplasts would be ideal sites in which to increase resistance to organic and inorganic Hg and repair damage resulting from Hg exposure [153]. 

Transgenic tobacco plants with engineered chloroplasts exhibited enhanced uptake of inorganic Hg, accumulating about 100-fold more than in untransformed plants [154]. An operon containing the bacterial genes *merA* (mercuric ion reductase gene) and *merB* (organomercurial lyase gene), expressed in tobacco chloroplasts, significantly improved plant tolerance to organic compounds [102]. Integrating the murine *Metallothionein* gene (*MT1*) into the tobacco chloroplast genome allowed high Hg accumulation within tobacco cells. These transplastomic lines were resistant up to 20 μM Hg and remained healthy with normal chlorophyll content and biomass [101]. Plastid transformation may also increase tolerance to high concentrations of copper, and sustain higher growth rates [91]. 

### 4.4. Biofuels Production

At present, biofuels research mainly focuses on the production of liquid fuel using sugars and lignocellulose from cassava (*Manihot esculenta*), sweet sorghum and other starchy or sugary non-grain crops as raw materials. The most important step in biofuels production is the hydrolysis of lignocellulose [155], with enzymatic digestion being the most efficient and environmentally friendly method, although fungi or bacteria producing the necessary cellulase make the process less efficient and more expensive. A chloroplast-based cellulolytic enzyme has been applied as an industrially pretreated feedstock (*Arundo donax*) for biofuel production [8]. The high levels and compartmentalization of toxic proteins possible within chloroplasts can however protect transgenic plants from multidirectional effects, turning the many chloroplasts within each cell into ideal bioreactors for industrial enzyme production [156]. Enzymes from various fungi and bacteria have been successfully produced in plant chloroplasts: for example, β-glucosidase [8,157], β-1,4-endoglucanase [158], cutinase, exoglucanase, pectinase, xylanase, lipase and acetyl xylan esterase were expressed in tobacco chloroplasts to produce fermentable sugars [111,159,160,161,162]. 

The enzymes derived from transplastomic plants have high activities for further applications. The β-glucosidase BglC and the endoglucanase Cel6A from *Thermobifida fusca* were highly active against synthetic test substrates when expressed in tobacco chloroplasts [157,163]. Treatment of cotton fiber with chloroplast-derived cutinase resulted in enlarged segments and the irreversible unwinding of intertwined inner fibers due to the expansion activity of cutinase. Transgenic plants accumulating cutinase also exhibited esterase and lipase activities [164]. A cocktail of these enzymes efficiently promoted sugar release from filter paper, pine wood and citrus peel [111]. The β-1,4-endoglucanase EGPh from the Archaeon *P. horikoshiiwere*, expressed in tobacco chloroplasts, can hydrolyze carboxymethyl cellulose (CMC) equally well in dry and fresh leaves. Furthermore, the inactive form of EGPh in mature leaves is easily removed by heat treatment [158]. The expression of endo-β-mannanase from *Trichoderma reesei* reached 25 units per gram of leaf (fresh weight), and the activity of endo-β-mannanase from chloroplast extracts was 6–7 fold higher than in *Echerichia coli* extracts, while also having higher temperature stability (40 °C to 70 °C) and wider pH optimum (pH 3.0 to 7.0) [149]. These reports provide convincing evidence that chloroplast-made enzymes have better temperature stability and a wider pH optimum range than those made in other systems [8,111,162].

The contributions of different elements of plastid transformation vectors can be assessed by comparing different efforts to produce the same protein. Xylanase is an important enzyme for lignocellulosic biomass fermentation and sugar release that has been transgenically expressed in tobacco. The xylanase genes used to date have been selected from different fungi or bacteria, expressed using different promoters, and inserted to the different sites in the chloroplast genome (Table 7). A comparison shows that the *Prrn* promoter drives the highest accumulation among these *xyn* transplastomic tobacco plants, although the *Prrn*-driven xylanase still has variable accumulation levels that might arise from the insertion site or source of the gene [8,111,150,161,165]. Kolotilin et al. tested different expression cassettes with *Prrn* or *psbA* promoter to express xylanase in tobacco [150]. Using *Prrn* as the promoter did indeed generate the most transcript in tobacco leave; however, it caused growth retardation in the transgenic plants, and ultimately produced similar protein accumulation levels as the construct using *psbA* as the promoter. These results indicate that mRNA accumulation can be too high in some cases and might even be lethal to the plant. 

Another factor to consider is that different sources of genes will have dissimilar codon usage. As the amount of foreign protein expressed in the chloroplast is related to both the promoter and its codon usage preference, codon optimization of the gene of interest provides an alternative way to increase protein expression [166].

### 4.5. Everything Looks Great, Right?

There are still many challenges facing the widespread adoption of plastid transformation technologies. These include: low transformation efficiency; lack of efficient screening methods for homoplasmy in transgenic plants outside of tobacco, especially in important crops such as rice, corn and other monocotyledonous plants, due to the lack of suitable selection markers and regulatory elements [167]; lack of appropriate tissue-specific regulatory sequences [168]; degradation of foreign proteins [169]; and foreign protein expression sometimes causing male sterility, yellow leaves and stunting. Inducible systems such as the ethanol-induced T7 promoter system, IPTG-lac system, and theophylline-inducible riboswitch system were developed because of the damage caused by constitutive foreign protein expression in host plants, but even these systems suffer from drawbacks: they are complex, toxic and costly. Although plastid transformation has been applied to various fields, it will also be critical to raise awareness among the general public of the usefulness of marker-free transplastomic plants.

## 5. Conclusions and Prospects

There is no doubt that plastid transformation has afforded a new direction for plant genetic engineering and constitutes a research hotspot because of its many advantages over nuclear transformation. Indeed, high transgene expression and engineering of polygenic traits are not amenable to classical nuclear transformation. However, plastid transformation can meet human needs, it is cost-effective, environmentally friendly, safe and efficient. It can be used to modify agronomic traits, and for phytoremediation and biofuels production. More importantly for human health, antigen vaccines and protein-based drugs can be produced in chloroplasts. Although plastid transformation has been achieved in many crops, many others still remain recalcitrant to plastid transformation [137]. The protocols of plastid transformation in crops would offer significant advantages for the production of edible vaccines and medical proteins, biofuels and industrial enzymes, as well as enhanced agronomic traits. In this review, we have illustrated the potential advantages from new studies and technologies like CRISPR-Cas9 for introducing double-strand DNA breaks for HR and create new varieties/mutants (*acc2*) to increase selection efficiency during plastid transformation. No longer limited to biolistic methods, nanotubes can be an alternative material for DNA delivery to increase the donor DNA into chloroplasts. Although they have not yet been applied to plastid transformation, the morphogenic regulators (*BBM*, *WUS2*, and cytokinin biosynthesis genes) and the new chemicals/technology for tissue culture and regeneration can increase the regeneration of transformed cells. These new approaches should also entice researchers to reconsider protoplast-based strategies for plastid transformation. Combined with nuclear transformation and other methods, plastid transformation may allow the production of important proteins. 

## Figures and Tables

**Table 1:** Species in which plastid transformation has been demonstrated.

Family	Scientific Name	Common Name	Selection Marker	Resistance	Method	Reference
Chlamydomonadaceae	*Chlamydomonas reindhartii*	Chlamydomonas	*aphA6*	Kan ^1^	Biolistic	[20]
Euglenaceae	*Euglena gracilis*	Euglena	*aadA*	Spec ^2^/Strep ^3^	Biolistic	[21]
Funariaceae	*Physcomitrella patens*	moss	*aadA*	Spec	PEG ^4^	[22]
Asteraceae	*Lactuca sativa*	lettuce	*aadA*	Spec	Biolistic	[23]
Amaranthaceae	*Beta vulgaris*	sugarbeet	*aadA*	Spec	Biolistic	[24]
Asteraceae	*Artemisia annua*	sweet wormwood	*aadA*	Spec	Biolistic	[17]
Brassicaceae	*Arabidopsis thaliana*	Arabidopsis	*aadA*	Spec	Biolistic	[25]
Brassicaceae	*Brassica capitate*	cabbage	*aadA*	Spec/Strep	Biolistic	[26]
Brassicaceae	*Brassica napus*	oilseed rape	*aadA*	Spec	Biolistic	[27]
Brassicaceae	*Brassica oleracea* var. *botrytis*	cauliflower	*aadA*	Spec	PEG	[28]
Brassicaceae	*Lesquerella fendleri*	popweed	*aadA*/*GFP*	Spec/Strep	Biolistic	[29]
Cucurbitaceae	*Momordica charantia*	bitter squash	*aadA*	Spec	Biolistic	[16]
Fabaceae	*Glycine max*	soybean	*aadA*	Spec	Biolistic	[30]
Malvaceae	*Gossypium* spp.	cotton	*aphA6/nptII*	KNO_3_/Kan	Biolistic	[31]
Poaceae	*Oryza sativa*	rice	*hpt*	Hygromycin	Biolistic	[32]
Salicaceae	*Populus alba*	poplar	*aadA*	Spec	Biolistic	[33]
Scrophulariaceae	*Scoparia dulcis*	licorice weed	*aadA*	Spec	Biolistic	[19]
Solanaceae	*Capsicum annuum*	pepper	*aadA*	Spec	Biolistic	[34]
Solanaceae	*Nicotiana tabacum*	tobacco	*aadA*	Spec	Biolistic	[8]
Solanaceae	*Solanum lycopersicum*	tomato	*aadA*	Spec	Biolistic	[35]
Solanaceae	*Scoparia melongena*	eggplant	*aadA*	Spec	Biolistic	[36]
Solanaceae	*Solanum tuberosum*	potato	*aadA*	Spec/Strep	Biolistic	[37]
Solanaceae	*Petunia xhybrida*	petunia	*aadA*	Spec/Strep	Biolistic	[38]
Umbelliferae	*Daucus carota*	carrot	*aadA*	Spec	Biolistic	[39]

^1^ Kanamycin; ^2^ Spectinomycin; ^3^ Streptomycin; ^4^ PEG-mediated transformations.

**Table 2:** Promoters, untranslated regions, and insertion sites commonly used for plastid transformation.

Promoters	5′-UTRs	3′-UTRs	Insertion Sites
*psbA*	*ggagg*	*psbA*	*trnl*/*trnA*
*rrn*	T7g10	*rps16*	*rbcL*/*accD*
*rbcL*	*rbcL*	*rbcL*	*trnfM-trnG*
	*psbA*	*petD*	*trnV*/*rps12*
	*atpB*		*trnN-trnR*
			*ycf3-trnS*

**Table 3:** Agronomic traits engineered into crops by plastid transformation.

Integration Site	Regulatory Sequence Promoter/Terminator	Transgene	Efficiency of Expression	Enhanced Trait(s)	References
*rbcL*/*accD*	*Prrn*/*rbcL* 3′	*panD*	>4-fold β-alanine	Photosynthesis and biomass production in response to high temperature stress	[90]
*trnI*/*trnA*	*T7g10* or *PpsbA*	*RbcS*	>150-fold RbcS transcript	Photosynthetic performance	[93]
*trnI*/*trnA*	*PpsbA*/*TpsbA*	*AQP1*, *TicAQP1*	16-fold transcript	Photosynthetic performance	[94]
*trnV*/*orf708*	*PpsbA*/*TpsbA*	*bicA*	~0.1% TLP	Photosynthetic performance	[95]
*trnV*/*rps12*	*Prrn*/*T7g10/Trps16*	*Trx f*, *Trx m*	700% leaf starch increased	Carbohydrate/starch content	[96]
*trnI*/*trnA*	*PpsbA*/*TpsbA*	*bgl-1*	>160-fold enzyme	Resistance to whitefly and aphids	[97]
*trnI*/*trnA*	*Prrn*/ggagg/*psbA 3′*	*tps1*	>169-fold transcript	Drought tolerance	[98]
*trnI*/*trnA*	*PpsbA*/*T7g10*/*Trps16*	*badh*	93–101 μmol/g DW	Salt tolerance (up to 400 mM NaCl)	[39]
*rbcL*/*accD*	*PpsbA*/*rbcL 3′*	*hppd*	5% TSP	Resistance to herbicide	[30]
*rbcL*/*accD*	*Prrn*/*TpsbA*	*EPSPS*	NR	Resistance to the herbicide glyphosate (>5 mM)	[99]
*rps7,12*/*trnV*	*Prrn*/*T7g10*/*Trps16*	*EPSPS*	>10% TSP	Resistance to the herbicide glyphosate	[92]
*trnV*/*rps12,7*	*Prrn*/*TrbcL*	*bar*	>7% in TSCP	Resistance to the herbicide phosphinothricin	[100]
*trnfM*/*trnG*	*PatpI*/*Trps16*	*Lycopene β-cyclase*, *Phytoene synthase*	NR	Herbicide resistance and carotenoid biosynthesis	[85]
*trnI*/*trnA*	*Prrn*/*T7g10*/*Trps16*	*mt1*	NR	Phytoremediation capability on mercury accumulation	[101]
*trnI*/*trnA*	*Prrn*/ggagg/*TpsbA*	*merA*, *merB*	NR	Phytoremediation capability on mercury accumulation	[102]
*trnI/trnA*	*PpsbA/TpsbA*	*RC101*, *PG1*	32–38% TSP; 17–26% TSP	Resistance to viral and bacterial infections	[103]
*trnI*/*trnA*	*Prrn*/*TpsbA*	*Bt-cry2Aa2*	45.3% TSP	Insecticidal protein content	[104]
*trnI*/*trnA*	*Prrn*/*T7g10*/*Trps16*	*MSI-99*	89.75 μg/g FW	Resistance to rice blast fungus	[105]
*trnV*/*rps12,7*	*Prrn*/*T7g10*/*TrbcL*	*cry1Ab*	NR	Resistance to caterpillar (*Anticarsia gemmatalis*)	[106]
*trnI*/*trnA*	*TrbcL*	*Bt-cry9Aa2*	~10% of TSP	Resistance to potato tuber moth (*Phthorimaea operculella*)	[107]
*rbcL*/*accD*	*Prrn*/*TpsbA*	*cry2Aa2*	2–3% TSP	Resistance to moth (*Heliothis virescens*, *Helicoverpa zea*, and *Spodoptera exigua*)	[108]
*rbcL*/*accD*	*PpsbA*/*SD*/*Trsp16*	*TC*, *γ-TMT*	3.05 nmol h^−1^mg^−1^ protein	Vitamin E content in tobacco and lettuce	[109]
*trnfM*/*trnG*	*Prrn*/*TrbcL*	*HPT*, *TCY*, *TMT*	NR	Vitamin E content in fruit; cold-stress tolerance	[35]
*trnI/trnA*	*Prrn/TpsbA*	*sporamin. CeCPI, chitinase*	0.85–1% TSP	Resistance to phytopathogens and insects	[89]
*trnI*/*trnA*	*PpsbA*/*TpsbA*	*cpo*	15-fold increased	Resistance to fungal infection (*Fusarium verticillioides*, *Verticillium dahliae* and *Alternaria alternata*)	[110]
*trnI*/*trnA*	*Prrn*/*T7g10*/*TpsbA*	*γ-TMT*	7.7% TLP	α-tocopherol content to regulate abiotic stress resistance	[91]
*trnI*/*trnA*	*PpsbA*/*TpsbA*	*PelB*, *PelD*	2.42 U/mg; 2.31 U/mg	Resistance to *Erwinia* soft rot	[111]
*trnI*/*trnA*	*PpsbA*/*TpsbA*	*pta*	5.16–9.27% TSP	Resistance to aphid, whitefly, Lepidopteran insects, and bacterial and viral pathogens	[112]
*trnI*/*trnA*	*PpsbA*/*TpsbA*	*phaA*	14.71 U/mg plant protein	Capacity for cytoplasmic male sterility engineering	[113]

DW: dry weight; FW: fresh weight; NR: not recorded; SD: Shine-Dalgarno sequence; TLP: total leaf protein; TSCP: total soluble cellular protein; TSP: total soluble protein.

**Table 4:** Vaccine antigens and protein-based drugs produced by chloroplasts.

Trait	Protein Being Expressed	Expression	Host Plant	References
Insulin	EX4	14.3% TSP	tobacco	[117]
Hemophilia B	FIX	1.79 mg/g DW in lettuce; 3.8% TSP in tobacco	lettuce; tobacco	[123] [124]
Hemophilia A	FVIII	852 μg/g DW in lettuce; 370 mg/g FW in tobacco	lettuce; tobacco	[23] [138]
Malaria	PMK, MVK, MDD, AACT, HMGS, HMGRt; IPP, FPP, ADS, CYP71AV1, AACPR	0.1 mg/g FW	tobacco	[125]
HIV	Pr55gag	78–% TSP	tobacco	[139]
HPV	E7	3–8% TSP	tobacco	[120]
Human cytokine	IFNα2b	3 mg/g FW	tobacco	[126]
Human cytokine	IFN-γ	6% TSP	tobacco	[140]
Human cytokine	hCT-1	5% TSP	tobacco	[141]
Cholera	AMA1	7.3 % TSP in tobacco; 13.2 % TSP in lettuce	tobacco; lettuce	[127]
Tuberculosis	Mtb72F and ESAT6	1.2–7.5% TSP	tobacco	[142]
Tuberculosis	CFP10, ESTA6 and dIFN	>0.035% TSP	carrot	[143]
Dengue virus	EDIII	0.8–1.6% TSP	tobacco	[144]

DW: dry weight; FW: fresh weight; NR: not recorded; TLP: total leaf protein; TSP: total soluble protein.

**Table 5:** Protein-based drugs produced in *Chlamydomonas reinhardtii* chloroplasts.

Therapeutic Protein	Expression	References
αCD22HCH23PE40, αCD22PE40 -Targets and kills B cell tumor	0.2–0.3% TSP	[20]
Tumor necrosis factor-related apoptosis-inducing ligand (TRAIL) -Leads to the apoptosis of cancer cells	0.43–0.67% TSP	[131]
GBSS-AMA1, GBSS-MSP1 -Anti-malarial	0.2–1.0 μg/mg Starch	[132]
Human glutamic acid decarboxylase (hGAD65) -For the treatment of Type I diabetes	0.25–0.3% TSP	[133]
Protein VP1 of Foot-and-mouth disease virus (FMDV-VP1) -Mucosal vaccine	3% TSP	[134]
Bovine mammary-associated serum amyloid (M-SAA) -Mucin induction	3–5% TSP	[135]
Protein E2 of classical swine fever virus (CSFV-E2) -Prevents classical swine fever	1.5–2% TSP	[136]
Protein VP2 of Infectious burial disease virus (IBDV-VP2) -Prevents IBDV infection	0.8–4% TCP	[137]

DW: dry weight; FW: fresh weight; NR: not recorded; TCP: total cellular protein; TSP: total soluble protein.

**Table 6:** Industrial enzymes and biomaterials obtained via chloroplast production in tobacco.

Products	Gene(s)	Expression	References
β-Glucosidase	*bgl-1*	44.4 U/g FW	[97]
β-Glucosidase, Cellulases	*bgl1*, *celA*, *celB*	9.9–58.2 U/mg of TSP	[147]
Cellulases, Xylanase	*endo*, *celB*, *xyn*	0.38–75.6% TSP	[8]
Cell wall-degrading enzyme	*bgl1C*, *cel6B*, *cel9A*, *xeg74*	5–40% TSP	[148]
β-Mannanase	*manI*	25 U/g FW	[149]
Xylanase	*xynA*, *xyn10A*, *xyn11B*	0.2–6% TSP	[150]
p-Hydroxybenzoic acid	*UbiC*	25% DW	[151]
Polyhydroxybutyrate	PHB pathway genes	18.8% TSP	[145]

DW: dry weight; FW: fresh weight; NR: not recorded; TCP: total cellular protein; TSP: total soluble protein.

**Table 7:** Xylanse produced by tobacco chloroplasts.

Gene	Source	Expression Level	Promoter	Insertion Site	Reference
*xyn*	*Alicyclobacillus acidocaldarius*	35.7% TSP	*Prrn*	rrn16/trnV–rps12/7	[8]
*xynA*	*Bacillus subtilis strain NG-27*	6% TSP	*Prrn*	rbcL-accD	[165]
*xyn2*	*Trichoderma reesei*	421 U/mg TSP	*Prrn*	trnI-trnA	[111]
*xyl10B*	*Thermotoga maritima*	13%TSP; 61.9 U/mg DW	*Prrn*	rbcL-accD	[161]
*xynA*	*Clostridium cellulovorans*	0.5% TSP	*Prrn* or *PpsbA*	trnI-trnA	[150]
*xyn10A*	*Aspergillus niger*	0.2% TSP	*PpsbA*	trnI-trnA	[150]
*xyn11B*	*Aspergillus niger*	6% TSP	*PpsbA*	trnI-trnA	[150]

DW: dry weight; TSP: total soluble protein.

## Abbreviations

CMC	Carboxymethyl Cellulose
CRISPR	Clustered Regularly Interspaced Short Palindromic Repeats
CSFV	Classical Swine Fever Virus
DAP	Day After Pollination
DSB	Double Strand Breaks
DW	Dry Weight
FW	Fresh Weight
GMO	Genetically Modified Organisms
HIV	Human Immunodeficiency Virus
HR	Homologous Recombination
IBDV	Infectious Burial Disease Virus
PEG	Polyethylene Glycol
SWNT	Single-Walled Carbon Nanotubes
TALEN	Transcription Activator-Like Effector Nucleases
TLP	Total Leaf Protein
TRAIL	Tumor Necrosis Factor Related Apoptosis-Inducing Ligand
TSP	Total Soluble Protein
TSCP	Total Soluble Cellular Protein
YFP	Yellow Fluorescent Protein
